# Spectral Distribution of Ultra-Weak Photon Emission as a Response to Wounding in Plants: An In Vivo Study

**DOI:** 10.3390/biology9060139

**Published:** 2020-06-26

**Authors:** Ankush Prasad, Prabhakar Gouripeddi, Hanumanth Rao Naidu Devireddy, Alina Ovsii, Dattatreya Prabhu Rachakonda, Roeland Van Wijk, Pavel Pospíšil

**Affiliations:** 1 Department of Biophysics, Centre of the Region Haná for Biotechnological and Agricultural Research, Faculty of Science, Palacký University, Šlechtitelů 27, 78371 Olomouc, Czech Republic; ovsijja@gmail.com (A.O.); pavel.pospisil@upol.cz (P.P.); 2 Sai Society for Advanced Scientific Research, Muddenahalli, Chikkaballapur 562101, Karnataka, India; gvprabhakar@ssasr.org (P.G.); hanumanth.naidu@ssslsg.org (H.R.N.D.); prabhu.rd@ssslsg.org (D.P.R.); 3 Sri Sathya Sai University for Human Excellence, Kalaburagi 585313, Karnataka, India; 4 Meluna Research, Koppelsedijk 1A, 4191 LC Geldermalsen, The Netherlands; roeland_van_wijk@meluna.nl

**Keywords:** ultra-weak photon emission, reactive oxygen species, Arabidopsis, oxidative radical reaction, wounding, mechanical injury, spectral properties

## Abstract

It is well established that every living organism spontaneously emits photons referred to as ultra-weak photon emission (synonym biophotons or low-level chemiluminescence) which inherently embodies information about the wellbeing of the source. In recent years, efforts have been made to use this feature as a non-invasive diagnostic tool related to the detection of food quality, agriculture and biomedicine. The current study deals with stress resulting from wounding (mechanical injury) on *Arabidopsis thaliana* and how it modifies the spontaneous ultra-weak photon emission. The ultra-weak photon emission from control (non-wounded) and stressed (wounded) plants was monitored using different modes of ultra-weak photon emission measurement sensors like charge-coupled device (CCD) cameras and photomultiplier tubes (PMT) and the collected data were analyzed to determine the level of stress generated, photon emission patterns, and underlying biochemical process. It is generally considered that electronically excited species formed during the oxidative metabolic processes are responsible for the ultra-weak photon emission. In the current study, a high-performance cryogenic full-frame CCD camera was employed for two-dimensional in-vivo imaging of ultra-weak photon emission (up to several counts/s) and the spectral analysis was done by using spectral system connected to a PMT. The results show that Arabidopsis subjected to mechanical injury enhances the photon emission and also leads to changes in the spectral pattern of ultra-weak photon emission. Thus, ultra-weak photon emission can be used as a tool for oxidative stress imaging and can pave its way into numerous plant application research.

## 1. Introduction

All living organisms including microorganisms, plants, and animals spontaneously generate ultra-weak photons which are synonymously referred to as biophotons or low-level chemiluminescence [1,2,3,4]. Ultra-weak photon emission is commonly recognized as photon emission with wavelengths ranging from near-ultraviolet to near-infrared (350–1300 nm) with intensity several orders lower than the sensitivity of the human eye. Although the intensity is extremely low, ultra-weak photon emission from living cells is well-known to possess information related to various types of physiological and/or pathological states in living systems [5,6].

The ultra-weak photon emission is closely related to various types of factors, such as exposure to exogenous toxins [7,8], pathogen attack [9,10], temperature fluctuations [11,12,13], acute stress [14,15,16,17], drought/flood [18], mechanical injuries [19,20,21], and exogenous reactive oxygen species (ROS) induced oxidative stress [5,22,23,24,25,26,27,28,29,30]. Thus, ultra-weak photon emission bears the potential to be employed in diagnostics of health-care, detecting the quality of food [31,32], and agriculture [8,17].

Ultra-weak photon emission results from reactions involving ROS formed during oxidative metabolic processes [25,33,34]. Reactive oxygen species generated at low concentrations in cells act as signaling molecules that facilitate significant responses in plant cells such as controlling growth, development and helps in developing tolerance to environmental strains [35,36,37]. On the contrary, at high levels they cause damage to cellular constituents triggering oxidative stress [38,39]. Whether the ROS produced would serve as signaling molecules or could cause oxidative damage to the plant tissues, depends entirely on the delicate equilibrium between ROS production, and their scavenging. Under continuous stress, ROS (superoxide anion and hydroxyl radicals, hydrogen peroxide and singlet oxygen etc.,) derived from molecular oxygen are accumulated in the leaves which then results in the oxidation of many critical cellular components including proteins, lipids, and nucleic acids. It can ultimately lead to the disruption of cellular metabolism activating the pathway of programmed cell death [40,41].

The emergence of a simple tool that can provide a physiological representation of oxidative stress would be a significant step toward monitoring this dynamic process in biological systems as well as improving our understanding of this process. Ultra-weak photon emission has been proposed as a potential tool for measuring oxidative processes [26,42,43]. The oxidative radical reactions have been extensively studied in recent years and have been proven to be closely associated with the formation of electronically excited species and subsequently to ultra-weak photon emission [43].

In the past decade, ultra-weak photon emission was based mainly on kinetic measurements using photomultiplier tubes (PMT) [30,42,44]. The kinetic measurements from the PMT augmented with filter system provides spectral data that could help in correlating photon emission from different spectral bands to the metabolic activities caused by the mechanical injury. In recent years, more promising charged coupled devices (CCD) cameras are being used for two-dimensional imaging that offers some exciting features in addition to kinetics such as high performance, extensive dynamic range, and low read-out noise [45,46,47,48,49,50]. A clear advantage of imaging is that it provides spatiotemporal information. In the current study, we have utilized ultra-weak photon emission as an indicator to differentiate between healthy and mechanically injured sites and to explore the spectral distribution of emitted photons.

## 2. Materials and Methods

### 2.1. Arabidopsis Plants and Leaves

Arabidopsis WT (Columbia-0) seeds were obtained from the Nottingham Arabidopsis Stock Centre (NASC), University of Nottingham (Loughborough, UK). The plants were between 6–7 weeks old grown under the following conditions: photoperiod, 8/16 h light/dark; photon flux density, 100 µmol photons m^−1^s^−1^; temperature, 22 °C/20 °C light/dark and relative humidity, 60%. Other conditions were as described in Prasad et al. 2019 [21]. For each measurement, a new Arabidopsis plant/leaf of the approximately same age and size was chosen.

### 2.2. Wound Treatment for Ultra-Weak Photon Emission Measurement

**Figure 1:**
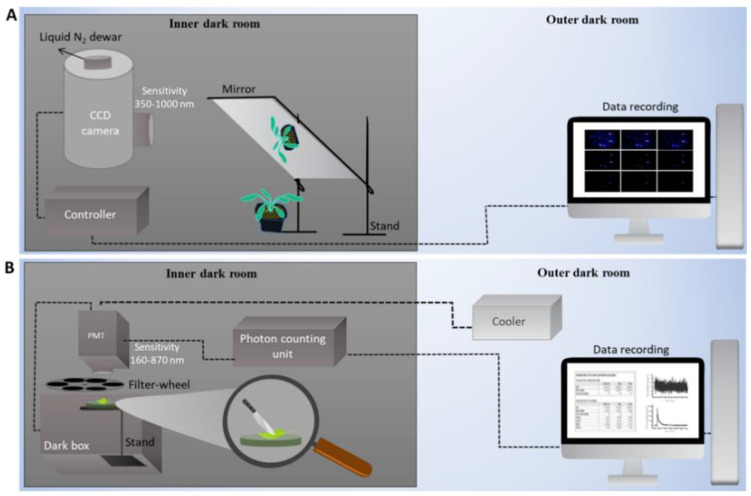
Schematic illustration of the experimental setup for two-dimensional imaging (**A**) and measurements of kinetics (**B**) of ultra-weak photon emission. The two-dimensional ultra-weak photon emission was measured using charge-coupled device (CCD) camera and the kinetic measurements of ultra-weak photon emission was measured using photomultiplier tubes (PMT) with a cut-on filter system.

Experiments were performed at room temperature and any interference from light sources and/or diffused light was completely avoided. Plants were dark-adapted under weak green light in the outer dark room for 2 h prior to measurements. Mechanical injury of Arabidopsis plant/leaf was carried out using a sharp razor blade (Figure 1). For CCD imaging, the mechanical injury was done in the presence of diffused green light with precaution so as not to exert pressure or damage other parts of the plant sample. The data accumulation was started 30 min after the mechanical injury for CCD measurements whereas it was kept 20 s for spectral measurements. CCD and PMT measurements were performed in replicates (*n* = 3 and *n* = 7, respectively).

### 2.3. Ultra-Weak Photon Emission

Ultra-weak photon emission was measured utilizing a highly sensitive CCD camera (VersArray 1300B, Princeton Instruments, USA). The CCD, which is a high-performance, full-frame, cryogenically cooled, scientific-grade camera has been configured with an objective lens and a mirror setup was created to direct the photon toward the objective (Figure 1A). Our overall system allows imaging of photon emission (to a level down to few photons s^−1^) from the biological system. The kinetic behavior and spectral analysis of photon emission were measured using PMT equipped with filter system PMT (9558QB; ET Enterprises Ltd., Uxbridge, UK). It was cooled to −28 °C with water-cooled thermoelectric housing (LCT50) to reduce the dark count. The photon emission signal was processed by the photon counting unit, MCS-CT3. A filter wheel housing was mounted between the PMT window and opening of the dark chamber (Figure 1B). The filter wheel contains 12 openings for 52 mm (2″) diameter cut-on absorption filters (Schott, Mainz, Germany) and was used for the spectral analysis of ultra-weak photon emission. Slots 1, 5, and 9 are empty and the remaining 9 slots contain filters having cut-on wavelengths 350, 400, 455, 495, 550, 610, 645, 695, and 780 (Table 1). Complete details of the filters and the filter system are given in Supplementary Materials, Table S1. The band emission (BE) was calculated using the formula

b_i_ = Max {0, f_i_ − 1 − f_i_}, for i = 1,...,10 (1)

where b_i_ is the emission from ith band, f_i_ is the emission measured from the ith cut-on filter, and f_10_ is the background noise. Shutter operation and the functioning of the filter wheel are executed by custom developed Visual Basic (VB) software. Because of the non-uniform quantum efficiency of PMT, the values measured from the filters can be corrected. However, we have not performed it in the current study as it makes only quantitative change and not qualitative.

### 2.4. Procedure for Spectral Measurement and Data Evaluation

For the setup used, the filter wheel rotates in steps letting each filter-slot stay under PMT for 8s and the emission count is recorded for each second giving 8 counts per second (cps) values/filter. In all calculations and representations, the average of 8 cps values referred to as filter-average or *f_avg* is used. Thus, there is one *f_avg* from each filter-slot when it is under the PMT for measurement. A complete rotation of the wheel (cycle) takes approximately 102 s [(9 slots with filters + 3 slots with no filters) × 8s + 4s for transition between slots]. To have a comprehensive data set, the BE (filter/band emission) and the total emission (TE) (no filters) data at the corresponding event specified by the pair (filter, cycle) were averaged over the seven leaves. The *f_avg* values from slots 1, 5, and 9 provide averaged TE and the *f_avg* values from the rest of the slots (1–4, 6–8, 10–12) provide averaged BE.

All spectral measurements are sequential in time, not simultaneous and the TE (no-filter) from spectral measurements is seen to decrease gradually with time (refer to section: Results). These two factors make a comparative study between bands and/or same band at different times difficult. To overcome this problem, we have computed the “interpolated total emission” (ITE) for each band measurement. This is achieved by constructing interpolation formula for each cycle using the four TE from no-filter slots 1, 5, and 9 of the cycle and first no-slot from the next cycle. Using this interpolation polynomial, ITE for each of the bands B1 to B10 was computed. The “percentage band emissions” (PBE) for each band was determined using the formula:

PBE = BE × 100/corresponding ITE(2)

## 3. Results

This section summarizes the results from the two different types of ultra-weak photon emission measurements described above arising from mechanical injury which is the core of the present investigation. The first part consists of CCD images demonstrating the spatial distribution of ultra-weak photon emission and the second part presents temporal spectral data, which embodies kinetics of ultra-weak photon emission.

### 3.1. Wounding and Ultra-Weak Photon Emission Imaging

**Figure 2:**
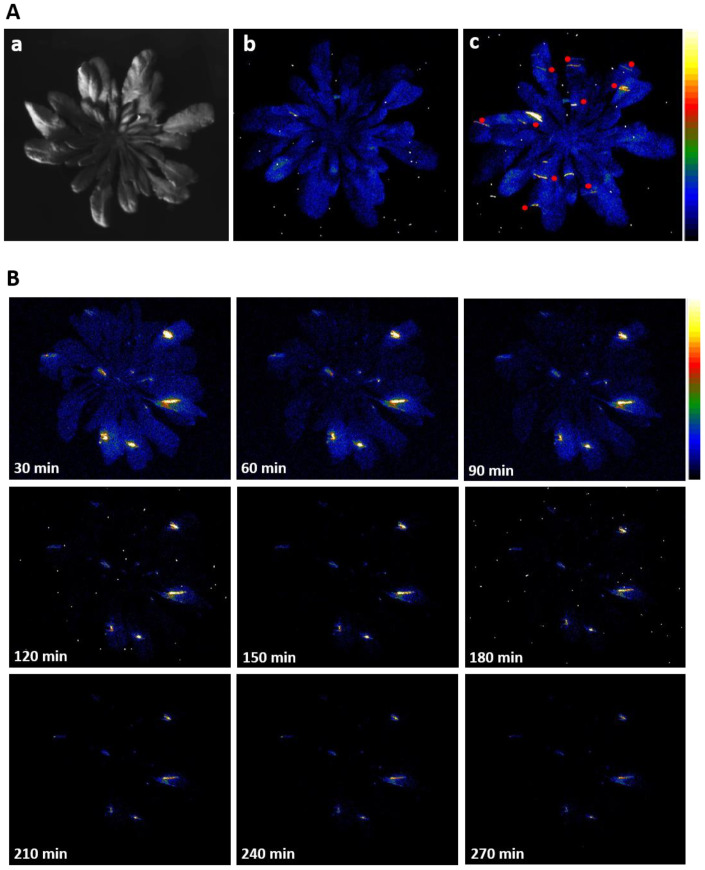
(**A**) Two-dimensional imaging of the ultra-weak photon emission from the Arabidopsis plants. The figure shows photographs (**a**) and the corresponding two-dimensional images of ultra-weak photon emission from non-wounded (**b**) and wounded (**c**) Arabidopsis plants. The plants were dark-adapted under weak green light for a period of 2 h before wounding. Mechanical injury was done under diffused green light and the sites are indicated by red dots within the image. The measurement was started 30 min after the mechanical injury with an accumulation time of 30 min. (**B**) Two-dimensional images of ultra-weak photon emission from wounded Arabidopsis plants under the condition described in (**A**) and subsequent images of the same plant were taken for 270 min each with 30 min accumulation time and variable dark adaptation (as indicated).

Arabidopsis plants subjected to mechanical injury was monitored by two-dimensional imaging of ultra-weak photon emission. Figure 2A shows a photograph (a) and two-dimensional images of ultra-weak photon emission measured in non-wounded (b) and wounded plant (c). Spontaneous ultra-weak photon emission observed from the non-wounded leaves of Arabidopsis is known to be contributed by metabolic oxidative processes while we can observe that the wounding of Arabidopsis leaves resulted in the enhancement of ultra-weak photon emission which is caused by wound-induced oxidative processes (red dots). It can be seen that high photon emission is solely restricted to the wounded part of the plant. It can also be seen that the ultra-weak photon emission following mechanical injury lasts for several hours (Figure 2B and Figure S2). It can be mentioned here that immediately following injury, there was an initial burst of oxidative radical reaction followed by propagative reaction continuing for several hours which resulted in higher intensity of ultra-weak photon emission.

### 3.2. Delayed Luminescence and Wound-Induced Ultra-Weak Photon Emission

**Figure 3:**
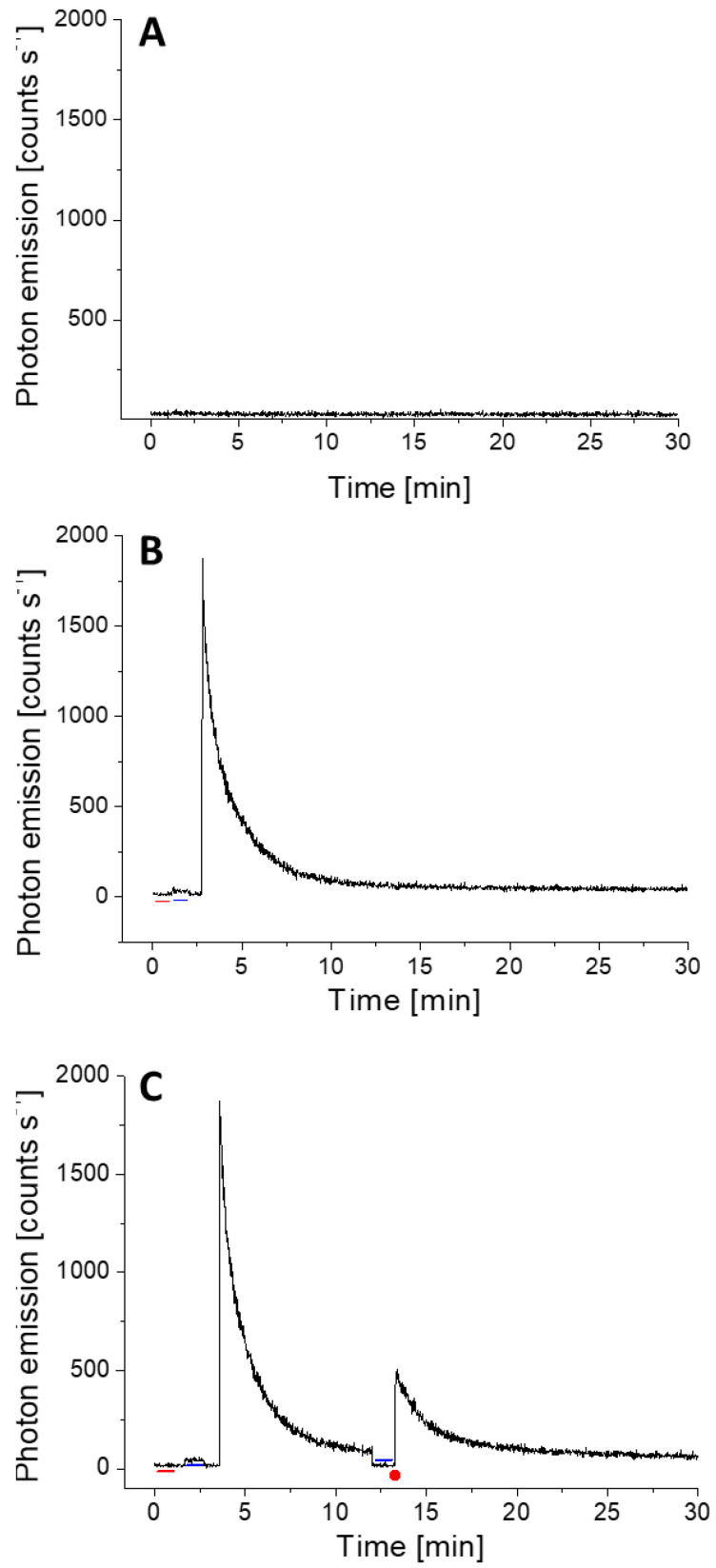
Mechanical injury-induced ultra-weak photon emission measured using PMT from Arabidopsis leaves. In (**A**), background level of photon emission was measured with an open shutter of the PMT. In (**B**), delayed luminescence from Arabidopsis leaf and its decay was monitored for 30 min. In (**C**), the delayed luminescence was measured for first 12 min followed by the kinetics of ultra-weak photon emission induced by mechanical injury (indicated by a red dot). In (**B**,**C**), the red and blue lines indicate the dark count and background noise, respectively. The decay curve in total was measured for 30 min.

The delayed luminescence, which is a light-induced photon emission, can last up to several minutes depending on various factors such as chromophore content of the sample, the intensity of exposed light, pre-adaptation to dark etc. In photosynthetic sample esp. Arabidopsis, we have monitored the phenomenon of delayed luminescence and it can last from a few up to a maximum of ~15 min. Figure 3A shows the background count of the PMT and Figure 3B shows the kinetics of delayed luminescence from 2 h dark-adapted Arabidopsis plants. It can be observed that the level of photon emission is close to spontaneous level after 10–12 min (Figure 3B). It was measured to estimate the time required for plants in complete dark to avoid any kind of interference from delayed luminescence.

**Figure 4:**
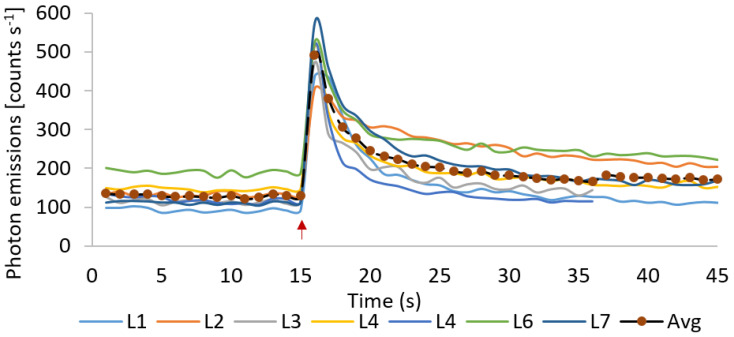
Mechanical injury-induced ultra-weak photon emission measured using visible PMT from seven different leaves (L1–L7) of Arabidopsis. Each plot is the average of *f_avg* TE values from no-filter slots 1, 5, and 9. *Y*-axis represents these average photon emission expressed as counts s^−1^. The *X*-axis represents time unit (34 s), which is the time gap between two consecutive no-filter (TE) measurements. Measurement was started ~20 s after mechanical injury indicated by the red arrow.

Following the standardization, the kinetics of ultra-weak photon emission was measured from Arabidopsis leaves subjected to mechanical injury. When wounding was done to Arabidopsis leaves after returning to the spontaneous level, it can be observed that the ultra-weak photon emission was enhanced to ~600 counts s^−1^ which then decayed over time (Figure 3C, Figures S3 and S4). The results were validated using seven different leaves (L1–L7), measured at a different time interval (Figure 4). Figure 4 shows that the mechanical injury immediately enhances the TE in all the cases and the average increase (black trace) was found to be 393 counts s^−1^ which was close to the values shown in Figure 3C. The increase in TE has two parts: an early transient part and a subsequent steady part. The transient part reached a steady-state after 8 min, which was 40 counts s^−1^ higher compared to the before injury part of emission. It remained in this state even after 15 min and possibly much longer as indicated by the CCD images. The state of the transient part could be viewed as oxidative burst due to mechanical injury while the steady photon emission can be a part of the recovery process.

### 3.3. Wounding and Spectral Distribution of Ultra-Weak Photon Emission

**Figure 5:**
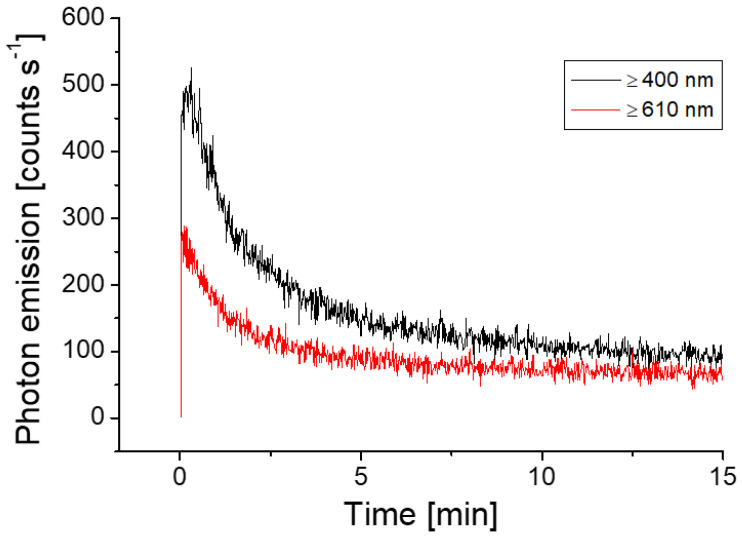
Kinetics of ultra-weak photon emission measured 20 s after the mechanical wounding in the presence of cut-on filters ≥400 nm and ≥610 nm. Other experimental conditions as described in Figure 3.

To understand the spectral distribution of ultra-weak photons emitted during wounding in Arabidopsis, we mounted cut-on absorption filters with transmission ranges ≥400 nm and ≥610 nm, respectively in the front of PMT window (Figure 1B). Kinetics of ultra-weak photon emission was measured from mechanically injured leaves. It can be observed that the ultra-weak photon emission in the spectral range of ≥400 nm is almost comparable to the value of ultra-weak photon emission without filter [Figure 3C, Figure 4 and Figure 5 (black trace)]. In addition, the contribution in the spectral range 600 nm and above, is approximately ~300 counts s^−1^ which is 50% of the corresponding value for ≥400 nm (Figure 5). The current observation indicates that not all ultra-weak photon emission observed during mechanical injury is contributed by chlorophyll but partially also comes from other electronically excited species emitting in the blue-green region. A partial reduction in photon counts, however, can be also attributed to percentage-transmittance of the filter and should be taken into consideration.

**Figure 6:**
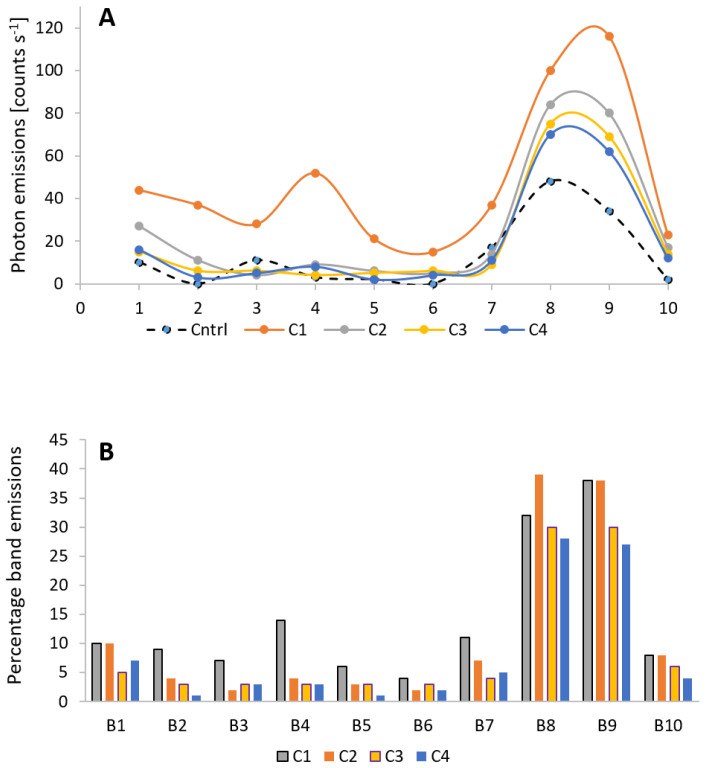
(**A**) The average photon emission from seven leaves expressed as counts s^−1^. A total of four cycles measured after mechanical injury presented as C1–C4 against the control (non-injured). The dots in 6A represent the BE (**B**). Band emissions (BE) [Equation (2)] obtained relative to interpolated total emission (ITE) for the first four cycles C1 to C4.

**Figure 7:**
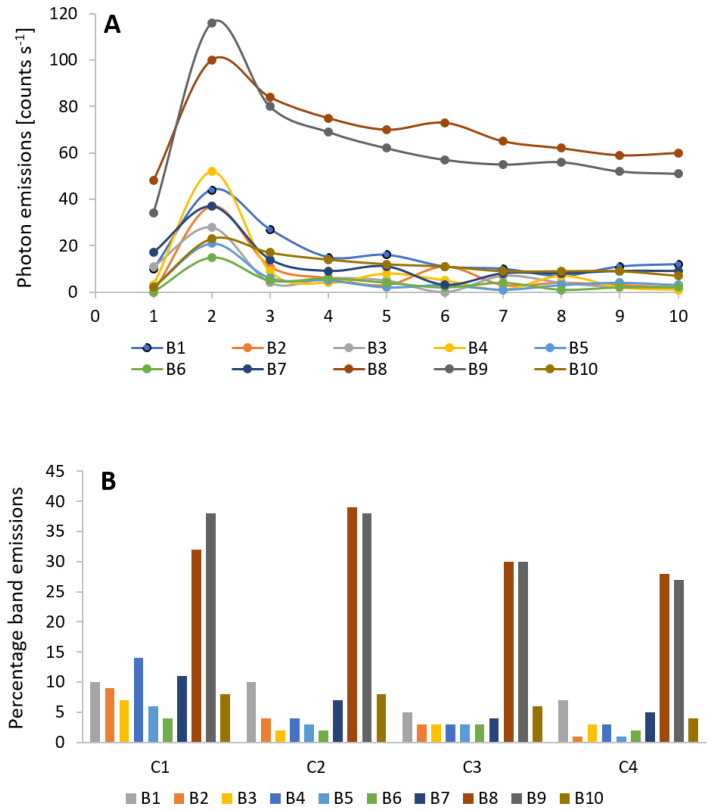
(**A**) Variation of average of BE (counts s^−1^) measured over a period of 10 cycles. (**B**) Percentage band emissions (PBE) [Equation (2)] obtained relative to ITE for four cycles after mechanical injury.

In addition to broad spectral studies, we also performed detailed spectral analysis following mechanical injury using a range of cut-on filters (Table 1). Figure 6 and Figure 7 present two perspectives of the data, namely BE w.r.t to cycles (Figure 6A,B) and w.r.t spectral bands (Figure 7A,B). The increase (above the control) is maximum in band B9 (116 counts s^−1^) followed by B8 (100 counts s^−1^) and the rest have values between 21 and 52 counts s^−1^. It is also interesting to note that band B4 which corresponds to spectral band 455–495 nm shows comparatively high photon emission in 1st cycle (C1). The ultra-weak photon emission in the next 102 s i.e., during C2 drops quickly by 68% in bands B2–B7 and by 28% in bands B1, B8–B10. There is a steady decrease in B8 and B9 and they do not return to spontaneous level before mechanical injury even after 15 min (a characteristic observed in TE). Statistical analysis using one-way analysis of variance (ANOVA) followed by Tukey-Kramer comparisons of band emissions (mean or raw data) coming from all the bands indicates that the photon emissions seen in bands B8 and B9 are significantly different from the rest of the bands at the 95% confidence interval. The significance of B1, B4, and B7 in the initial stage was also confirmed by this analysis. The photon emissions in other bands decrease at different rates and they rather fluctuate and remain close to control state after 10 min (Figure 4).

### 3.4. Oxidative Stress, Electronically Excited Species, and Ultra-Weak Photon Emission

Based on the spatial distribution as observed in Figure 2, it can be seen that injured sites show the higher intensity of ultra-weak photon emission which indicates a high level of oxidative radical reaction at these sites. The overall ultra-weak photon emission observed at the site of wounding can be attributed to ROS produced as a consequence and successive oxidative radical reactions which led to the generation of electronically excited species. The ultra-weak photon emission observed in Figure 5 (red trace) can be attributed to the photon emission from excitation energy transfer from triplet excited carbonyl (^3^R=O*) to chlorophylls and/or to molecular oxygen forming ^1^O_2_.

## 4. Discussion

Stress-induced alterations in plants have been mainly detected after destructive sampling followed by biochemical and molecular determinations. Techniques that allow immediate detection of oxidative stress level, before visual symptoms appear and adverse effects become established can be of immense advantage. Ultra-weak photon emission techniques using photon counting or imaging are emerging as promising tools for crop yield management [14]. The currently available technology allows ultra-weak photon emission measurement only under laboratory conditions in total darkness. Therefore, any research in this direction until now requires designing experiments by simulating the stress conditions and on a small scale. In most investigations, the physiological and chemical changes brought by different stresses are correlated with the ultra-weak photon emission, statistics, and image patterns thereby predicting that the changes in ultra-weak photon emission are capable of capturing and assessing these stress conditions. The present investigation is one more step in this direction. It has been designed to study the effects of abiotic stress (wounding) and its relation to ultra-weak photon emission. It has been possible to pool our resources and to investigate the mechanical injury using both CCD imaging and PMT based spectral analysis. Correlating the results from the two approaches is the attempt here.

Mechanical injury is, among many other stress conditions, explored using the ultra-weak photon emission. Wounding can directly disrupt physiological function and are potential infection sites for opportunistic pathogens [21,51]. Recent studies have revealed that ROS might play an important role in inducing protection mechanisms during both biotic and abiotic stresses. Consequently, the avenues of anti-oxidant production and its regulation have become an active research field. Mechanical injury-induced ultra-weak photon emission in Spathiphyllum sp. leaves under aerobic and anaerobic conditions was recently studied in the visible spectrum (300–720 nm) [52]. The authors have presented results that the mechanical injury-induced ultra-weak photon emission was significantly suppressed when under anoxic stress. It was shown that photon emission is in the spectral region >650 nm, implicating chlorophyll as the likely emitter, which is in agreement with results obtained for Arabidopsis in the present investigation (Figure 5) [53,54]. Ultra-weak photon emission and ^1^O_2_ generation in germinating soybean in response to wounding were observed using a high-sensitivity imaging system based on an intensified charge-coupled device and a highly sensitive single photon counter by Chen and co-workers [14]. The ultra-weak photon emission intensity of wounded cotyledons was initially very high and reached a stationary state after few min which also agrees with our observation (Figure 4). The fast-kinetic drop in the first few min of mechanical injury may represent an oxidative burst which could be a result of the trauma caused by the mechanical injury. A more stable phase observed after about 10 min of wounding can be explained as an indication of the recovery process from oxidative stress (Figure 2, Figure 3, Figure 4 and Figure 5). Based on the observation that the emission was suppressed in the presence of sodium azide and deuterium oxide which amplified the photon emission intensity, the authors concluded that ^1^O_2_ is the main candidate involved in photon emission which also agrees with our confocal imaging studies using singlet oxygen sensor green that showed the formation of ^1^O_2_ at the site of mechanical wounding [21].

The generation of ultra-weak photon emission as a response to wounding comes from the oxidation of biomolecules such as lipids, protein, and nucleic acid. Based on the results obtained from oxidized linoleic/linolenic acid, it was presented that a major contribution of photon emission was in the red region (>600 nm) [23,54] while only 20% of TE was observed <600 nm [23,54]. From the spectral measurements (Figure 4, Figure 6, Figure 7 and Table S5), a computation corroborates the above statement. It was observed that for different wavelength the decay kinetics seem to be different (Figure 5); sometimes a rapid decay (B4), sometimes slow (B8–10). It was observed that the band 645–780 nm (bands B8 and B9) contributes to 70% of TE in first 102 s [cycle C1] and reaches a maximum of 77% during the next 102 s [cycle C2] and then decrease to 55% and below subsequently. Band B4 (455–495 nm) was initially 14% and drops to 4% or less by cycle C2.

Ultra-weak photon emission associated with lipid peroxidation and protein oxidation is usually attributed to ^3^R=O* and ^1^O_2_, which are by-products of decomposition of reactive intermediates such as dioxetanes and tetraoxides [55]. Relaxation of ^3^R=O* is known to emit in the range of 350–550 nm while dimol emission from ^1^O_2_ occurs at 634/703 nm. However, because of the presence of chlorophyll in plants, the efficient energy transfer results in more significant photon emission in the range of 670–740 nm and thus a major contribution is seen. Nevertheless, in some non-photosynthetic sample, it has been shown that major photon emission is in the region 450–800 nm, thus we can suggest that chlorophyll is not an exclusive candidate of photon emission in the green-red region [33]. Our investigation clearly show a relatively high photon emission in band B4 compared to neighboring bands during the cycle C1. From the data presented in Figure 4, Figure 5, Figure 6 and Figure 7, it can be clearly stated that a major enhancement of ultra-weak photon emission as a response to wounding is in the red region of the spectrum and includes nearly 70% of the TE, but the underlying reason is most likely the presence of chlorophylls in the close proximity [43,54] or other species emitting in the red region of the spectrum. For optimal measurements of other regions of the spectrum of ultra-weak photon emission, the sample should not contain chlorophyll. Makino and co-workers [56] previously reported a strong photon emission during the interaction between *Fusarium oxysporum* and the sweet potato storage root which does not contain chlorophylls. This system is also suitable for continuous spectral analysis of emission. They showed shifts in spectra in time, in parallel with several kinds of enzymatic activities related to ROS generation.

## 5. Conclusions

Wounding does produce an increase in TE leading to increase in the all BE which rapidly drop with time and reach a new steady-state higher than the spontaneous emission of pre-wounding. The induced BE are not uniform with respect to the spectral pattern between band which in turn points toward potential alterations in metabolism. After a sufficiently long time, which is of the order 10–15 min, ultra-weak photon emission reaches a quasi-steady state and the metabolism seems to reach a steady-state of recovery, leaving the system at a higher energy state of equilibrium. All these observations are qualitatively reflected in the CCD images. We believe that a combination of transcriptomics, proteomics, and metabolomics with ultra-weak photon emission can bring a new, broader view about the spectral shift of ultra-weak photon emission in response to wounding and enabling the application of ultra-weak photon emission as an efficient, reliable, and non-invasive method for monitoring the state of a living organism.

## Figures and Tables

**Table 1:** The band emission labels (b1–b10) and the range of their transmission (in nm) for all filters used in the spectral analysis.

Band Emission	b1	b2	b3	b4	b5
Band range (nm)	<350	350–400	400–455	455–495	495–550
Band emission	b6	b7	b8	b9	b10
Band range (nm)	550–610	610–645	645–695	695–780	>780

## Supplementary Materials

The following are available online at https://www.mdpi.com/2079-7737/9/6/139/s1, Table S1: Table S1 gives the arrangement of filters on the filter wheel, their corresponding cut-on wavelength and the labels used for denoting the corresponding cut-on emissions. Figure S2: Time-lapse video showing the ultra-weak photon emission from Arabidopsis plants subjected to mechanical wounding taken over 210 min with a 30 min accumulation time for each frame (total images: 7). The plants were dark-adapted under weak green light for a period of 2 h before the measurement. Mechanical injury was done under weak green light and plant was further kept in complete darkness for 30 min before measurement. Figure S3: Delayed luminescence from Arabidopsis leaf and its decay monitored for 30 min. The data show the level of dark count and background noise of the PMT (grey box). The arrow indicates the point at which the PMT shutter was closed to assure that the level of noise does not change with time. Figure S4: Biological replicates showing the fluctuation in delayed luminescence. The red dot in A and B indicates mechanical injury. The grey box in B shows opening and closing of the dark box during the measurement to make sure that no external noise was created during the process of wounding. All other conditions remain as in Figure 3C. Table S5: Table S5 showing raw data used in Figure 6 (red) and Figure 7 (blue). The data show averaged BE and TE derived as average from the seven leaves selected for analysis. The first row is the control data, the data from the cycle just before the mechanical injury. Rows C1–C9 are the data from the nine cycles after the mechanical injury. Each entry in the table is the average of the corresponding data of seven selected leaves. The data marked as red in the first table were used for Figure 6A and the one marked blue in the table two were used for Figure 7B.
