# Supplementary material for: Spectral Distribution of Ultra-Weak Photon Emission as a Response to Wounding in Plants: An In Vivo Study
Source: Biology (Basel). 2020 Jun 26;9(6):139. doi: 10.3390/biology9060139 (PMC7345010; doi:10.3390/biology9060139)
Supplement: Supplementary file 1 [file biology-09-00139-s001.zip › biology-819047-supplementary.pptx]

## Slide 1
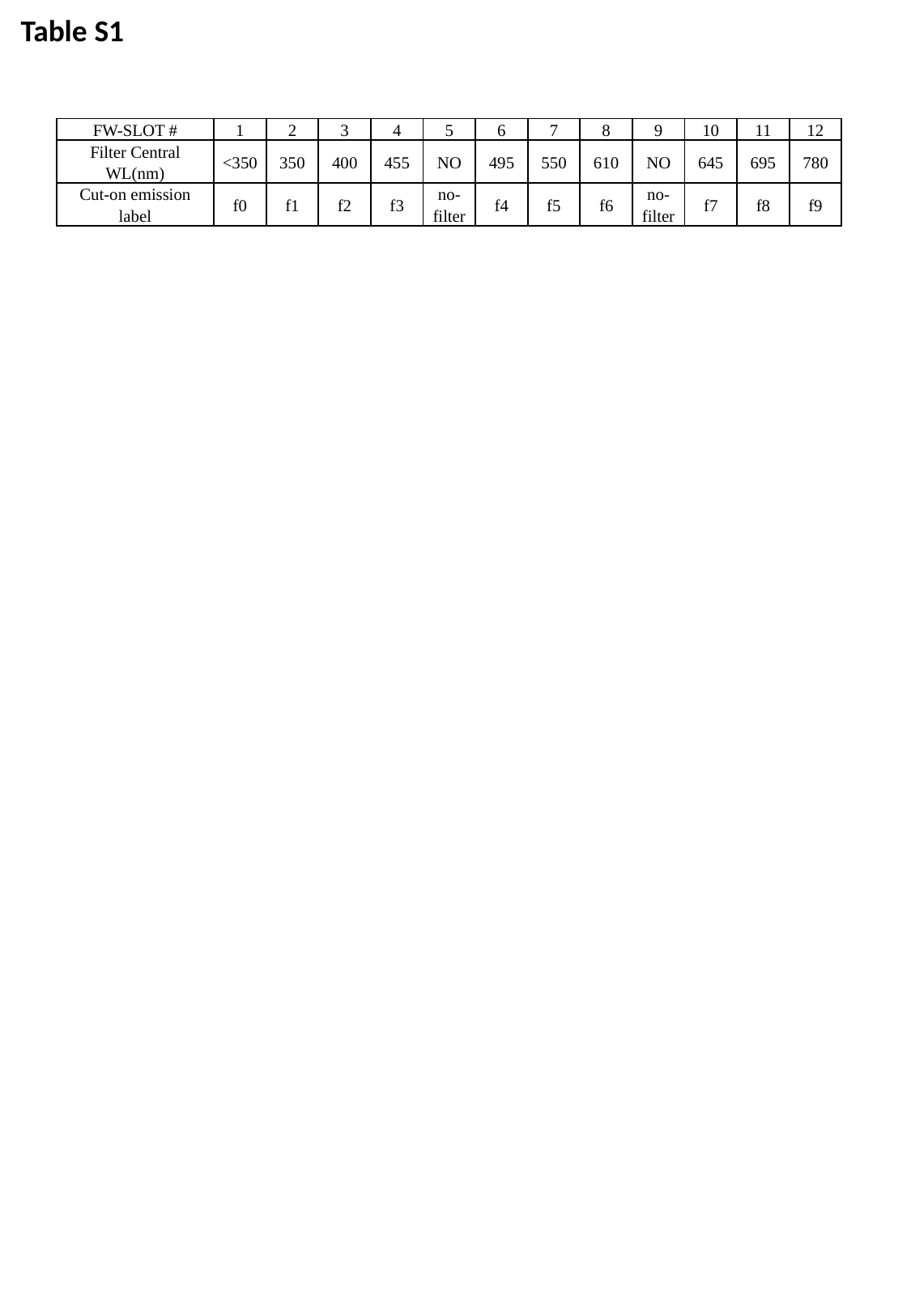

Table S1
| FW-SLOT # | 1 | 2 | 3 | 4 | 5 | 6 | 7 | 8 | 9 | 10 | 11 | 12 |
| --- | --- | --- | --- | --- | --- | --- | --- | --- | --- | --- | --- | --- |
| Filter Central WL(nm) | <350 | 350 | 400 | 455 | NO | 495 | 550 | 610 | NO | 645 | 695 | 780 |
| Cut-on emission label | f0 | f1 | f2 | f3 | no-filter | f4 | f5 | f6 | no-filter | f7 | f8 | f9 |

## Slide 2
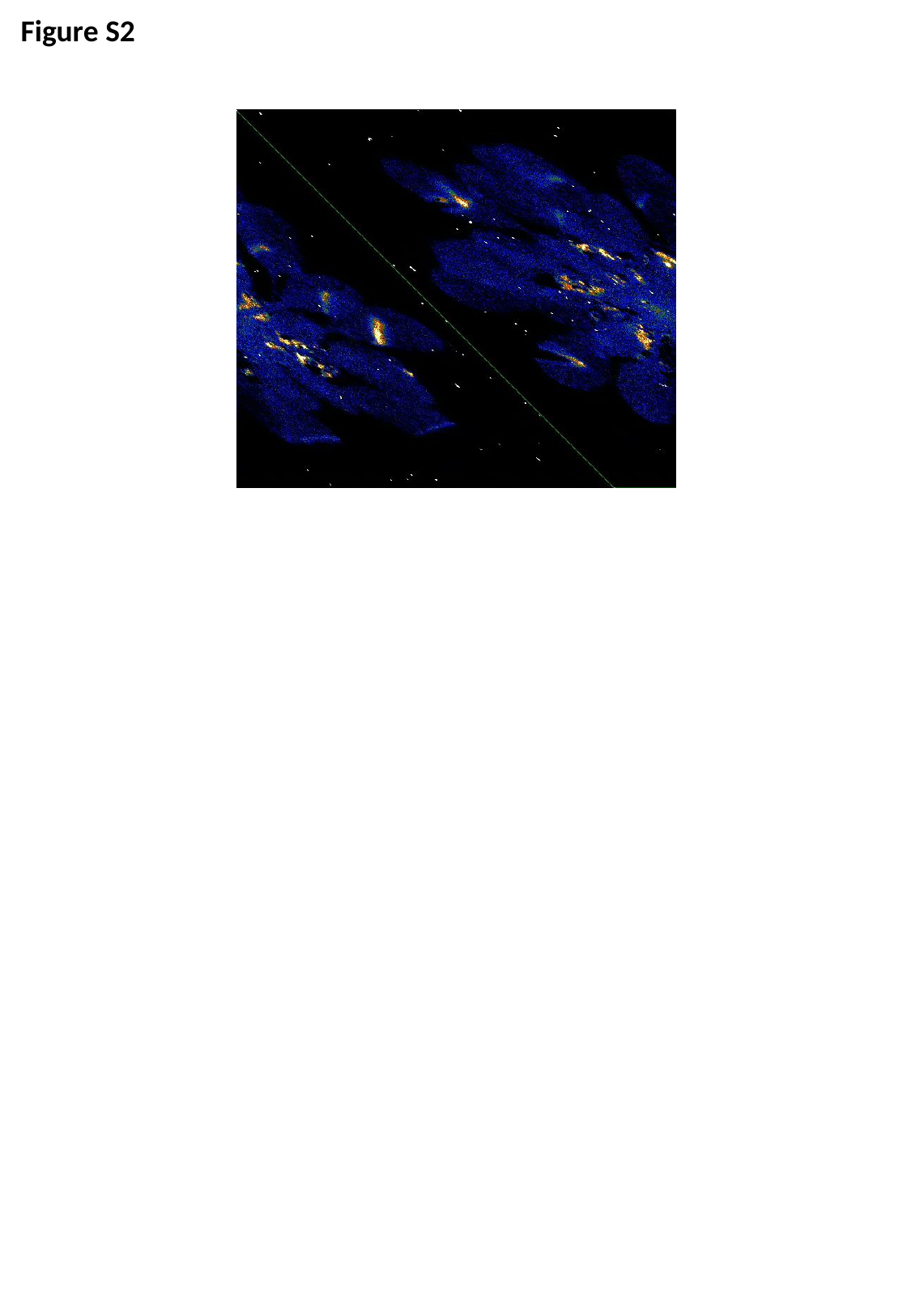

Figure S2

## Slide 3
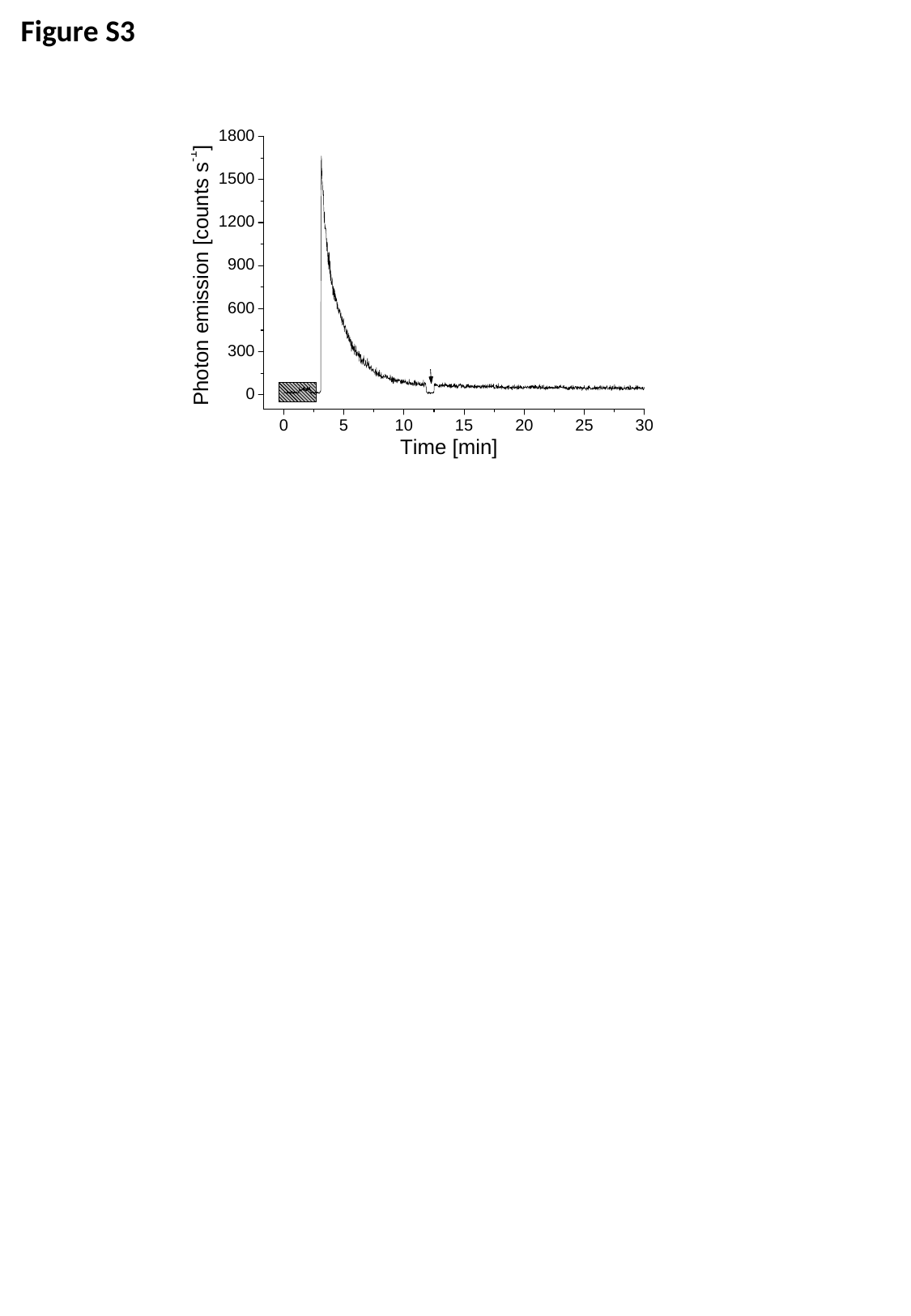

Figure S3

## Slide 4
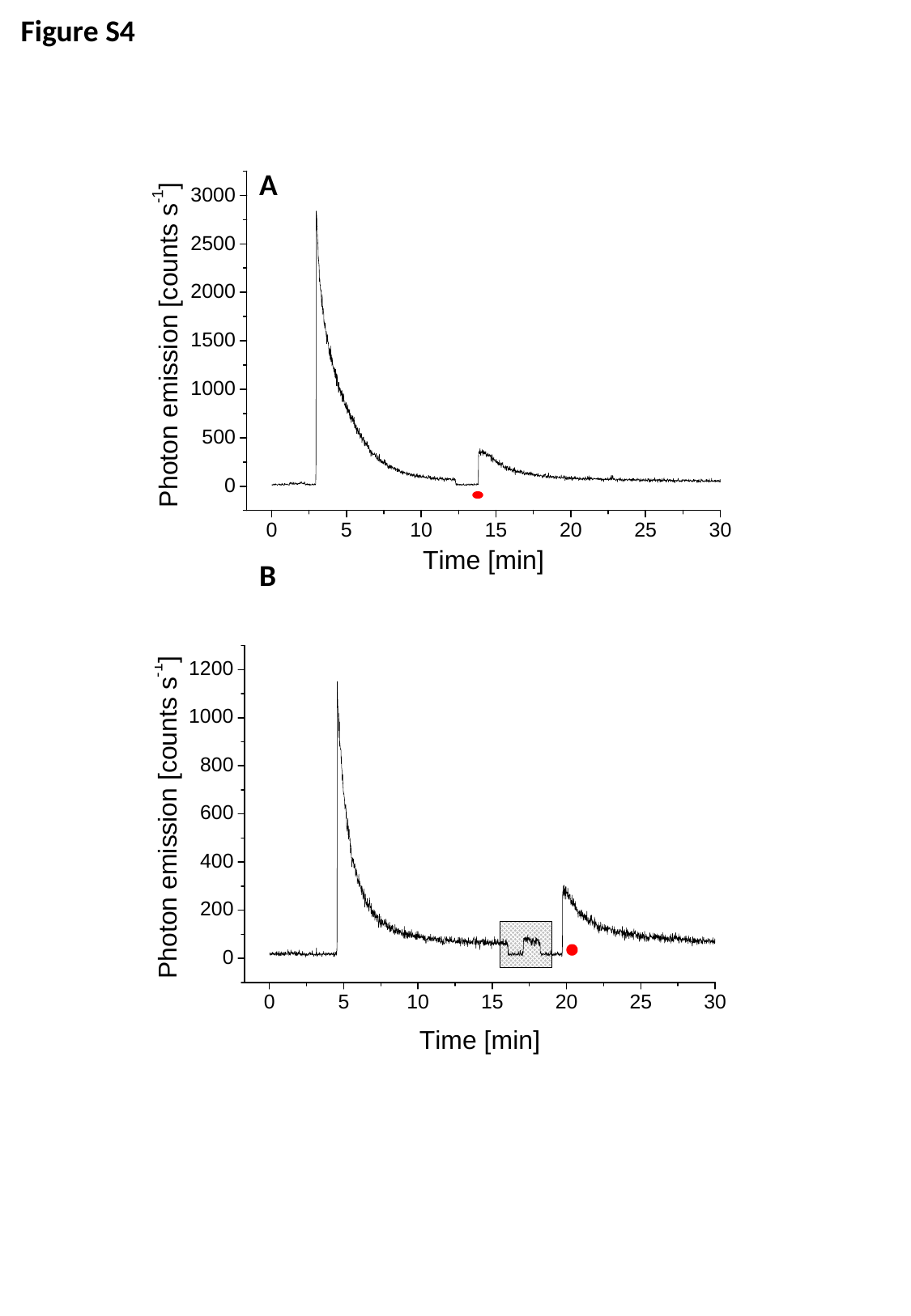

Supplementary data 2
Figure S4
A
B

## Slide 5
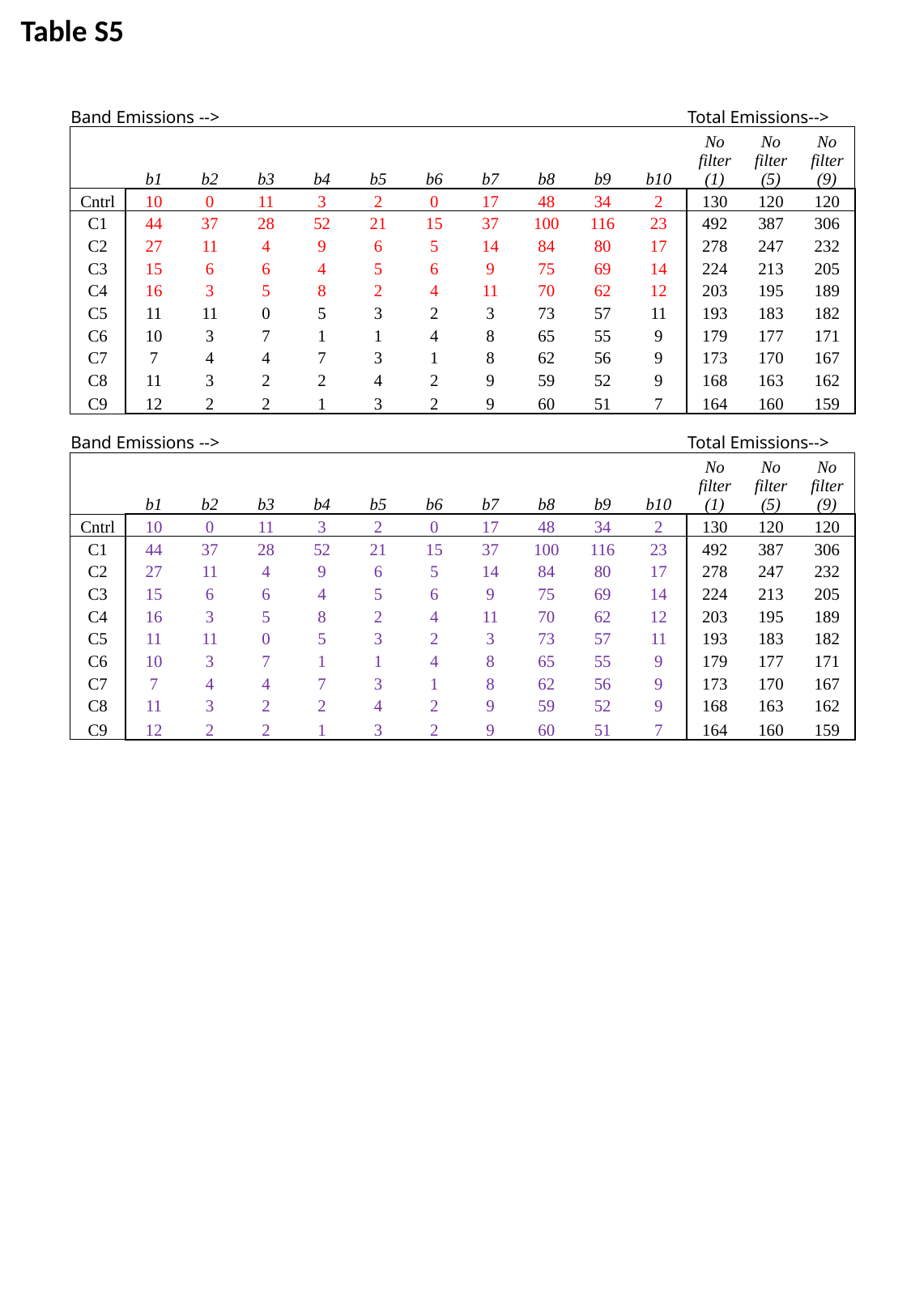

Table S5
| Band Emissions --> | | | | | | | | | | | Total Emissions--> | | |
| --- | --- | --- | --- | --- | --- | --- | --- | --- | --- | --- | --- | --- | --- |
| | b1 | b2 | b3 | b4 | b5 | b6 | b7 | b8 | b9 | b10 | No filter (1) | No filter (5) | No filter (9) |
| Cntrl | 10 | 0 | 11 | 3 | 2 | 0 | 17 | 48 | 34 | 2 | 130 | 120 | 120 |
| C1 | 44 | 37 | 28 | 52 | 21 | 15 | 37 | 100 | 116 | 23 | 492 | 387 | 306 |
| C2 | 27 | 11 | 4 | 9 | 6 | 5 | 14 | 84 | 80 | 17 | 278 | 247 | 232 |
| C3 | 15 | 6 | 6 | 4 | 5 | 6 | 9 | 75 | 69 | 14 | 224 | 213 | 205 |
| C4 | 16 | 3 | 5 | 8 | 2 | 4 | 11 | 70 | 62 | 12 | 203 | 195 | 189 |
| C5 | 11 | 11 | 0 | 5 | 3 | 2 | 3 | 73 | 57 | 11 | 193 | 183 | 182 |
| C6 | 10 | 3 | 7 | 1 | 1 | 4 | 8 | 65 | 55 | 9 | 179 | 177 | 171 |
| C7 | 7 | 4 | 4 | 7 | 3 | 1 | 8 | 62 | 56 | 9 | 173 | 170 | 167 |
| C8 | 11 | 3 | 2 | 2 | 4 | 2 | 9 | 59 | 52 | 9 | 168 | 163 | 162 |
| C9 | 12 | 2 | 2 | 1 | 3 | 2 | 9 | 60 | 51 | 7 | 164 | 160 | 159 |
| Band Emissions --> | | | | | | | | | | | Total Emissions--> | | |
| --- | --- | --- | --- | --- | --- | --- | --- | --- | --- | --- | --- | --- | --- |
| | b1 | b2 | b3 | b4 | b5 | b6 | b7 | b8 | b9 | b10 | No filter (1) | No filter (5) | No filter (9) |
| Cntrl | 10 | 0 | 11 | 3 | 2 | 0 | 17 | 48 | 34 | 2 | 130 | 120 | 120 |
| C1 | 44 | 37 | 28 | 52 | 21 | 15 | 37 | 100 | 116 | 23 | 492 | 387 | 306 |
| C2 | 27 | 11 | 4 | 9 | 6 | 5 | 14 | 84 | 80 | 17 | 278 | 247 | 232 |
| C3 | 15 | 6 | 6 | 4 | 5 | 6 | 9 | 75 | 69 | 14 | 224 | 213 | 205 |
| C4 | 16 | 3 | 5 | 8 | 2 | 4 | 11 | 70 | 62 | 12 | 203 | 195 | 189 |
| C5 | 11 | 11 | 0 | 5 | 3 | 2 | 3 | 73 | 57 | 11 | 193 | 183 | 182 |
| C6 | 10 | 3 | 7 | 1 | 1 | 4 | 8 | 65 | 55 | 9 | 179 | 177 | 171 |
| C7 | 7 | 4 | 4 | 7 | 3 | 1 | 8 | 62 | 56 | 9 | 173 | 170 | 167 |
| C8 | 11 | 3 | 2 | 2 | 4 | 2 | 9 | 59 | 52 | 9 | 168 | 163 | 162 |
| C9 | 12 | 2 | 2 | 1 | 3 | 2 | 9 | 60 | 51 | 7 | 164 | 160 | 159 |
